# Tamsulosin-Induced Atrial Fibrillation With Rapid Ventricular Response

**DOI:** 10.7759/cureus.25714

**Published:** 2022-06-07

**Authors:** Duncan McGuire, Heaveen Ahdi, Nicholas Mielke, Amit Bahl

**Affiliations:** 1 Emergency Medicine, Beaumont Health, Royal Oak, USA; 2 Emergency, Oakland University William Beaumont School of Medicine, Royal Oak, USA

**Keywords:** case report, benign prostatic hypertrophy, arrhythmia, atrial fibrillation, tamsulosin

## Abstract

Tamsulosin, sold under the brand name Flomax, is a commonly prescribed medication for benign prostatic hypertrophy (BPH). While generally well-tolerated, this medication can induce life-threatening tachyarrhythmias. Early recognition of this adverse effect can lead to prompt discontinuation of the therapy in an effort to reduce arrhythmia recurrence. Herein we describe a case of atrial fibrillation with rapid ventricular response in a male patient who started on tamsulosin two days prior.

## Introduction

Atrial fibrillation is a common arrhythmia with a prevalence of 2.3%-3.4% and a lifetime risk of one in four [1]. Overall, recurrence rates are as high as 40%-50%, and up to 17% of patients requiring cardioversion return to the emergency department (ED) within 30 days of the initial onset [2-4]. These recurring episodes of atrial fibrillation increase the disease burden significantly and contribute to the development of serious adverse sequelae, including stroke, heart failure, and even cardiac arrest when not treated appropriately and promptly [2,3]. The most common precipitants of atrial fibrillation include surgery and pneumonia. Rarer causes include medication side effects, cardiovascular disease, respiratory illness, metabolic disorders, and illicit drug use [1,2,5].

One commonly used medication that can induce cardiac arrhythmias is tamsulosin (trade name Flomax). This medication is prescribed as a first-line agent for symptomatic benign prostatic hypertrophy (BPH), a condition that affects an estimated three out of every four men greater than 70 years old [6]. While it is generally well tolerated with limited serious side effects, post-marketing reports have identified atrial fibrillation as a rare but potentially life-threatening adverse effect of tamsulosin. Further, in a summary of adverse event data on clinical pharmaceutical trials of tamsulosin, atrial fibrillation was reported to occur in 0.1%-0.6% of patients [7,8]. Given this drug’s widespread use, providers should consider this etiology in patients taking this medication who develop or have a recurrence of atrial fibrillation. Prompt recognition and discontinuation of the instigating medication will allow for appropriate medical management while reducing the likelihood of arrhythmia recurrence. We describe a case involving a patient who experienced atrial fibrillation with rapid ventricular response two days after starting tamsulosin.

## Case presentation

A 61-year-old male presented to an academic tertiary care emergency department with a chief complaint of acute onset palpitations and light-headedness that began earlier that morning after standing quickly. Prior to the onset of these symptoms, the patient was in his usual state of health. He denied associated chest pain or pressure, shortness of breath, recent alcohol intake, or illicit drug use. Of note, his primary care provider (PCP) started him on tamsulosin 0.4 milligrams daily two days prior for newly diagnosed BPH. 

**Figure 1:**
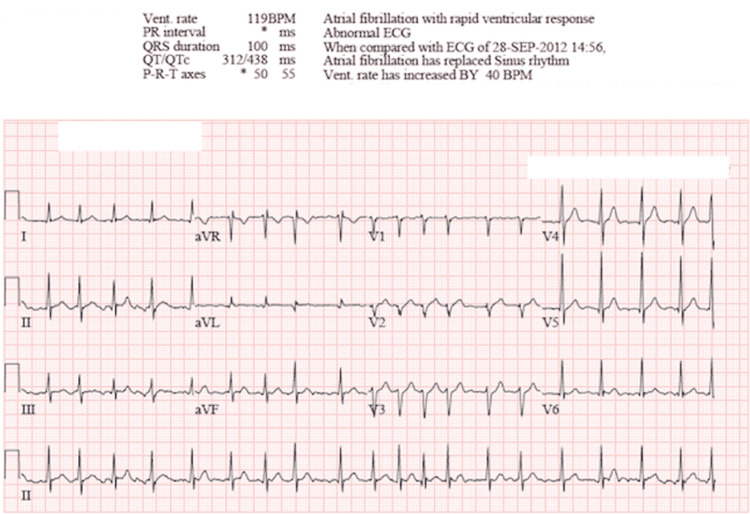
Initial ECG obtained in the emergency department demonstrating atrial fibrillation with rapid ventricular response

Upon arrival at the emergency department, the patient was hemodynamically stable with a blood pressure of 168/78, heart rate of 117 beats per minute, respiratory rate of 17 breaths per minute, blood oxygen saturation of 100% on room air, and was afebrile with a temperature of 97.9°F (36.6°C). On physical exam, the patient was a well-groomed, non-toxic male weighing 90.7 kilograms who appeared his stated age. The only remarkable finding was an irregularly irregular tachycardic rhythm appreciated on cardiac auscultation and palpation of extremity pulses. An electrocardiogram (ECG) demonstrated an irregularly irregular narrow complex tachycardia consistent with atrial fibrillation with the rapid ventricular response (Figure 1).

**Figure 2:**
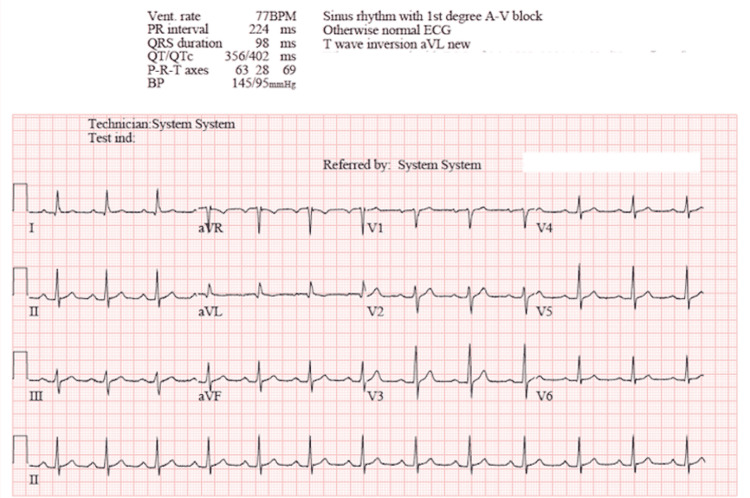
Electrocardiogram obtained following synchronized cardioversion demonstrating sinus rhythm.

Laboratory analysis including complete blood count, basic metabolic panel, magnesium, phosphorous, troponin I (TnI), thyroid-stimulating hormone and free T4 were within the normal reference ranges per the hospital system’s electronic medical record. The patient underwent electrical cardioversion, successfully converting the arrhythmia to sinus rhythm (Figure 2).

Upon waking from sedation, the patient expressed resolution of his symptoms. After performing a literature review, tamsulosin was identified as a precipitant of atrial fibrillation. Given that the patient was started on tamsulosin two days prior and that there were no other clearly identified etiologies of this arrhythmia, the case was discussed with both the patient's PCP and cardiologist. It was agreed upon that the patient was stable for discharge with the instructions to temporarily discontinue tamsulosin until further outpatient evaluation occurred. The patient was started on apixaban therapy and directed to follow up with his PCP and cardiologist for continued management.

## Discussion

Tamsulosin is a common adrenergic alpha-1 receptor antagonist used in the treatment of BPH. The frequency of its use is attributed to its efficacy and safety, which stems from its rather specific mechanism of action. Unlike other alpha-1 adrenergic antagonists, tamsulosin selectively binds alpha-1A/D receptors located on the prostate rather than the alpha-1B receptors present in vasculature [9]. The medication inhibits detrusor muscle contractions in the prostate, thereby reducing resistance to urinary flow and alleviating voiding problems [9].

Despite this high selectivity, tamsulosin is associated with potential side effects. The class of adrenergic alpha-1 antagonists was originally designed to treat hypertension, and many of the adverse effects associated with tamsulosin use are associated with alpha-a1 receptor-mediated vasodilation [9]. Clinical trial data demonstrate a causal relationship between tamsulosin and dizziness, headache, orthostatic hypotension, palpitations, and reflex tachyarrhythmias [9]. Atrial fibrillation has been reported as an exceedingly rare dose-dependent side effect in two post-marketing clinical trials, the largest of which noted two cases out of the 2,152 patients on tamsulosin [7,8]. Both patients, who were taking tamsulosin 0.8 mg daily, had this medication discontinued after developing this arrhythmia [7]. 

**Figure 3:**
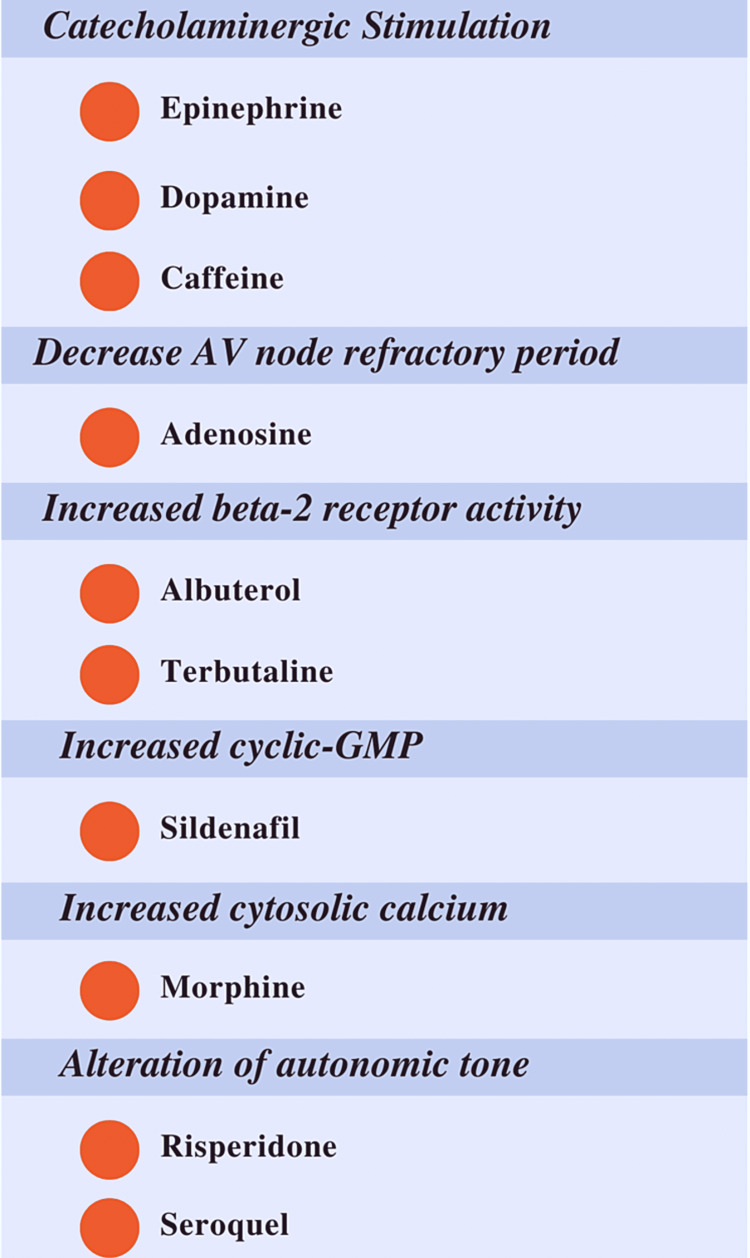
Causes of medication-induced atrial fibrillation Mechanisms and medications responsible for precipitating atrial fibrillation

There are several mechanisms responsible for medication-induced atrial fibrillation, varying based on the culprit medicine. These pathways may involve catecholaminergic stimulation, a shortened atrial refractory period, increased beta-2 receptor activity, increased cyclic-GMP or cytosolic calcium, and alterations in autonomic tone (Figure 3) [1]. In the case of this patient, the alpha-a1 mediated decrease in autonomic tone was believed to result in a hypotension-induced reflex tachycardia which triggered atrial fibrillation requiring cardioversion. Ultimately, without cardiac ablation, patients have nearly a 70% chance of atrial fibrillation recurrence within four years, and those with a prior history of tachyarrhythmias are even more prone to medication-related atrial fibrillation [1,10].

Aside from brief summary trial data, there is currently sparse evidence describing details of tamsulosin-induced atrial fibrillation. While atrial fibrillation is a frequently encountered condition in the emergency room, the root cause of the arrhythmia is not always clear. However, identifying the instigating etiology may yield tailored treatment strategies and improved patient outcomes. By recognizing tamsulosin as a cause of orthostatic hypotension, which can induce reflex tachyarrhythmias, emergency medicine physicians may not only play a role in the acute management but also the recurrence of this arrhythmia. This can be done by informing the patient of this potential medication side effect and providing instruction to discuss this relationship with their prescribing doctor. If the medication is continued, the patient should be given resources to monitor their heart rate and blood pressure at home in the event that the arrhythmia recurs [4]. Additionally, as more is learned about the relationship between tamsulosin and hypotension-induced tachyarrhythmias, healthcare providers should prescribe tamsulosin cautiously to patients with a prior history of hypotension and atrial fibrillation. There are hemodynamically neutral medications, like the 5-alpha-reductase inhibitor finasteride, that can be prescribed for BPH and should be considered by outpatient physicians managing this condition [7].

## Conclusions

Tamsulosin is an alpha-a1 antagonist commonly used to treat symptoms associated with BPH. Through alpha-a1 mediated vasodilation, tamsulosin-induced orthostatic hypotension may result in reflex tachyarrhythmias, including atrial fibrillation. As first-line providers for patients experiencing atrial fibrillation and given the widespread use of the medication, it is important for emergency medicine providers to be wary of this side effect. This knowledge will enable emergency physicians to not only acutely manage the arrhythmia, but also aid in the continued evaluation and management of this patient by primary care, cardiology, and urology services.
